# Immunohistochemical Investigation of HER/AKT/mTOR Pathway and Cellular Adhesion Molecules in Urothelial Carcinomas

**DOI:** 10.1155/2017/6794150

**Published:** 2017-01-22

**Authors:** Nikolaos Koletsas, Triantafyllia Koletsa, Spyros Choidas, Konstantinos Anagnostopoulos, Stavros Touloupidis, Thomas Zaramboukas, Georgia Raptou, Nikolaos Papadopoulos, Maria Lambropoulou

**Affiliations:** ^1^Urology Department, Interbalkan Medical Center, Thessaloniki, Greece; ^2^Pathology Department, Medical School, Aristotle University of Thessaloniki, Thessaloniki, Greece; ^3^Laboratory of Biochemistry, School of Medicine, Democritus University of Thrace, Alexandroupolis, Greece; ^4^Urology Department, School of Medicine, Democritus University of Thrace, Alexandroupolis, Greece; ^5^Laboratory of Histology-Embryology, School of Medicine, Democritus University of Thrace, Alexandroupolis, Greece

## Abstract

*Background*. Several investigators have suggested the possibility that the expression of both EGFR and HER2 could be utilized for molecularly targeted therapy in urinary bladder cancer. We tried to evaluate the expression of HER2 and EGFR and activation of the AKT/PTEN/mTOR pathway in urothelial carcinomas and if there is any association between them and cellular adhesion molecules (CAMs).* Materials and Methods*. Forty-one paraffin-embedded urothelial cancer tissue blocks were collected. Immunostains for HER2, EGFR, MIB1, phospho-AKT, PTEN, phospho-mTOR, e-cadherin, p-cadherin, and b-catenin were performed on tissue microarrays sections. The immunohistochemical results were correlated with clinicopathological parameters.* Results*. The overexpression of HER2 was found in 19.6% of the cases and it was associated with high grade tumors with a high mitotic index and phosphorylation of AKT and mTOR. Muscle-invasive tumors presented both cytoplasmic and nuclear losses of PTEN expression. There was no association between HER/AKT/mTOR pathway activation and CAM expression. Although cadherins were often coexpressed, only p-cadherin immunoreactivity was associated with tumor grade and high proliferative index.* Conclusions*. HER2 overexpression is found in a respective proportion of urothelial carcinomas. P-cadherin expression is associated with high grade UCs but it is not affected by HER2 overexpression or by activation of HER/AKT/mTOR pathway.

## 1. Background

The human epidermal growth factor receptors (HER) protein family consists of four different transmembrane receptors (HER1–HER4). HER1/EGFR and HER2/c-erb-B2 are the most thoroughly investigated family members and have been documented to be involved in the pathogenesis of several types of cancers. In urothelial carcinomas HER1 and HER2 expression has been implicated in tumor aggressiveness, poor outcome, or even pathogenesis [1–4]. In recent years, their importance has been emphasized due to the development of targeted anti-HER therapy.

The dimerization of HER members leads to the activation of intracellular RAS/MEK/ERK [5] and PI3K/AKT/PTEN/mTOR [6, 7] pathways which plays an important role in cell proliferation, angiogenesis, invasion, and metastasis. The PI3K/AKT/PTEN/mTOR pathway is considered to be essential for cell growth, survival, cell motility, and angiogenesis [8–11]. The activation of this pathway has been implicated in carcinogenesis or malignant potential of several cancers, including urothelial ones [12, 13].

The categorization of urothelial carcinomas is based on grading and muscle invasion. The majority of urothelial carcinomas are noninvasive tumors of low grade [14]. Muscle invasion carcinomas are characterized by mutations in TP53, RB1, and PIK3CA genes and deletions in PTEN gene [4, 15] and loss of e-cadherin [16]. Cell adhesion molecules (CAMs) are required for maintaining a normal epithelial phenotype and abnormalities in their expression have been related to cancer progression [16].

The present study was conducted to investigate the expression of EGFR and HER2 proteins, as well as intracellular signaling molecules in sections of urothelial carcinomas by immunohistochemistry, to analyze e-cadherin, p-cadherin, and b-catenin expression in low and high grade urothelial carcinomas, and to correlate the immunohistochemical results with clinicopathologic parameters.

## 2. Materials and Methods

A total of 41 archived cases of urothelial bladder carcinomas were included in this study. Clinical data and complete follow-up were known in 23 patients. The pertinent hematoxylin and eosin (HE) stained sections were retrieved and reevaluated by pathologist and the representative neoplastic areas corresponding to tumor classification and grading were marked for tissue microarrays formation.

### 2.1. Construction of Tissue Microarrays (TMAs)

Formalin-fixed paraffin-embedded (FFPE) tissue samples from urothelial tumors (paraffin blocks) were collected retrospectively. TMA blocks were constructed with the Alphelys Minicore 3 Tissue Microarray system (Plaisir, France). Each tumor was represented by 3 tissue cores, 1 mm in diameter, which were obtained from the marked representative areas of neoplasms and reembedded in recipient paraffin blocks. TMAs also contained cores from placenta, tonsil and normal thyroid, breast, and renal and colon tissue, used as control markers and for section orientation. Four-micrometer-thick sections were obtained and stained by immunohistochemical (IHC) method.

### 2.2. Immunohistochemistry

IHC staining was performed on freshly cut sections. Primary antibodies against HER2 (polyclonal, Dako, Glostrup, Denmark), EGFR (clone 31G7, Invitrogen, Carlsbad, CA, USA), phospho-AKT 1/2/3 (Thr308)-R (polyclonal, Santa Cruz Biotechnology, Santa Cruz, CA, USA), PTEN (clone 6h2.1, Dako, Denmark), phospho-mTOR (Ser2448) (clone 49F9, Cell Signaling Technology, Danvers, MA, USA), Ki67 (clone MIB1, Dako, Denmark), E-cadherin (#610181, BD Transduction Laboratories, San Jose, CA, USA), and beta-catenin (#610153, BD Transduction Laboratories, San Jose, CA, USA) were used. IHC stains were performed on a Bond automated stainer (Dako).

### 2.3. Immunohistochemical Evaluation

There is no standard protocol or guidelines for the estimation of HER2 expression in urothelial carcinomas or what the most appropriate cutoff value is. The HER2 immunostain scoring was performed based on the guidelines of the American Society of Clinical Oncology/College of American Pathologist (0: no staining, 1+: incomplete membranous staining, 2+: complete but weak or moderate membranous staining in >10% of cells, and 3+: strong membranous staining in more than 10% of the cells) [17]. There is no standard protocol for EGFR evaluation. EGFR expression was considered as positive when complete membrane positivity was observed in a percentage of >10% of the cells, as it was used before [18]. For phospho-AKT (pAKT) and PTEN both percentage of positive cells and intensity for nuclear and cytoplasmic immunoreaction were evaluated. The percentage of positive tumor cells (0–100%) was multiplied by dominant staining intensity (1: weak, 2: medium, and 3: intense) and a cutoff value based on the median tumor *H*-score was used, as described by Gonzalez-Roibon et al. [19]. phospho-mTOR (pmTOR) immunostaining was considered negative when expression was observed in <10% of cells and positive if immunoreactivity was found in ≥10% of cells [20]. A tumor was considered to have a high mitotic index when there were positive cells to Ki67/MIB1 antibody in a percentage of >20%.

In 35 cases with adequate specimen immunohistochemistry for CAM expression was also applied. Eighteen out of 35 tumors were of high grade while only ten were invasive. Tumors with positive cells in a percentage >10% was considered positive for e-cadherin, p-cadherin, and b-catenin. According to the intensity of staining tumors were categorized as weak, moderate, and strong [21].

### 2.4. Statistical Analysis

The statistical software package SPSS v. 21 was used for statistical analyses. Chi-square test was employed to test the dependence between different parameters. Values of 0.05 or less were considered to be statistically significant.

## 3. Results

### 3.1. Clinicopathological Characteristics of Patients

Forty-one patients were included in the study, 32 males and 9 females. Their mean age was 68 years (range 47–87). Tumor characteristics are presented in Table 1. Nineteen of the carcinomas were low grade (46.3%) while 22 (53.7%) were of high grade. All tumors with advanced stage were of high grade. Almost one-third of the cases were muscle-invasive tumors. Patients with stages pTa and pT1 were treated either with epirubicin or BCG depending on histological grade, tumor size, and multiplicity. Radical cystectomy was followed for those patients with invasive tumors. Chemotherapy was added in two cases with metastatic disease. A third patient underwent only radiotherapy due to the small size of a solitary lesion located on the frontal bladder wall.

### 3.2. Immunohistochemical Distribution of the Markers

**Figure 1:**
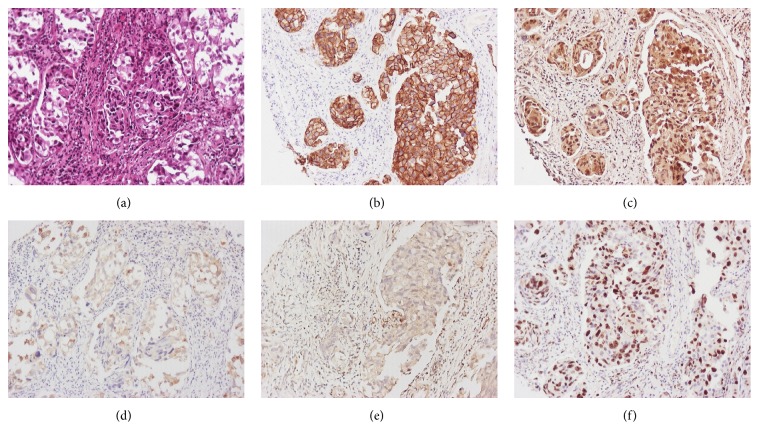
A case of high grade infiltrative urothelial carcinoma (a) presenting HER2 overexpression (b), pAKT (c) and pmTOR (d) expression, loss of PTEN expression (e), and high MIB1 labeling index (f). ((a) HE ×200; (b–f) IHC ×200).

The immunohistochemical results are summarized in Table 2. HER2 overexpression (3+) was found in 8 cases (19.6%), seven of which were of high grade (*p* = 0.032). HER2 2+ and 3+ immunoscores accounted for almost 46.4% and were mainly found in high grade tumors (*p* = 0.179). Sixty percent of HER2 3+ tumors measured more than 3 cm (*p* = 0.039). HER2 expression exhibited positive correlation with pAKT cytoplasmic and nuclear immunoreactivity (*p* = 0.049 and *p* = 0.021, resp.) (Figure 1). Moreover, in 12 out of 19 HER2 positive cases, pmTOR was coexpressed (*p* = 0.021). The majority of the HER2 positive cases had high mitotic index (*p* = 0.021), defined as >20% positive cells to Ki67/MIB1 antibody.

HER2/EGFR coexpression was observed in four cases (9.75%). There was no association between EGFR expression (14/41, 34.14%) or HER2/EGFR coexpression (4/41, 9.75%) and the examined clinicopathologic parameters (*x*^2^, *p* > 0.05).

Fifteen out of eighteen high grade cases (83.3%) presented high mitotic indices (*p* = 0.001). MIB1 positivity was associated with HER2 positivity (*p* = 0.021) and pmTOR cytoplasmic (*p* = 0.035) expression (*p* < 0.001).

Loss of PTEN cytoplasmic expression was found mainly in muscle-invasive tumors (*p* = 0.001) (Table 3). A loss of PTEN expression was defined as simultaneous lack of nuclear and cytoplasmic immunoreactivity. Muscle-invasive tumors presented commonly a loss of PTEN expression (*p* = 0.023). None of the cases without cytoplasmic PTEN staining exhibited cytoplasmic expression of pAKT (*p* = 0.032). PTEN cytoplasmic expression was positively associated with the cytoplasmic expression of pmTOR protein (*p* = 0.01). However, lack of PTEN nuclear immunoreactivity was not associated with any of the other studied markers, apart from a trend of negative association observed with pAKT nuclear expression (*p* = 0.09). In three cases PTEN was immunoreactive in membranes, as well.

The majority of the muscle-invasive tumors (pT2–pT4) (9/13, 69.2%) expressed pmTOR protein compared to pTa-pT1 urothelial carcinomas (*p* = 0.045) (Table 3). Cytoplasmic pmTOR expression was associated with high MIB1 labeling index (*p* = 0.035) and neoplastic invasion (*p* = 0.045). Notably, membranous immunoreactivity to pmTOR was found in seven cases.

In this cohort, HER2 overexpression along with pAKT nuclear expression, both nuclear and cytoplasmic PTEN deletion and pmTOR expression, was found in three of the patients. Two of them coexpressed the EGFR protein, and they had the worst prognosis.

**Figure 2:**
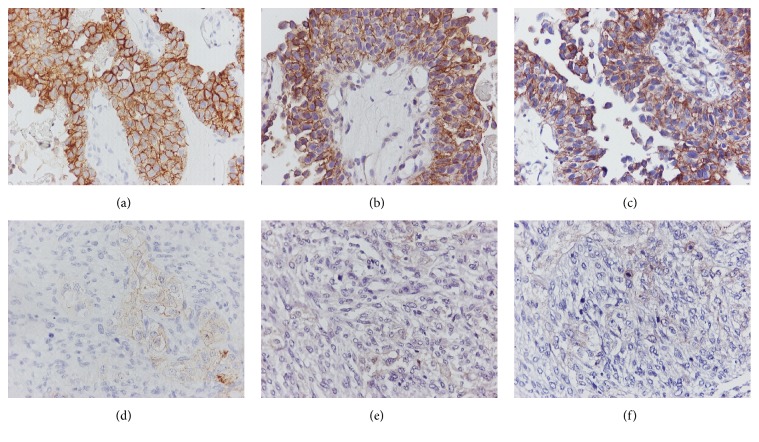
CAM expression in different urothelial tumors. A case of noninfiltrative high grade urothelial carcinoma positive for e-cadherin (a), p-cadherin (b), and b-catenin (c) markers, in contrast to high grade infiltrative urothelial carcinoma with sarcomatoid features, exhibited no or weak expression in few cells to e-cadherin (d), p-cadherin (e), and b-catenin (f) antibodies ((a–f) IHC ×400).

Expression of e-cadherin and p-cadherin was observed in 54.3% (19/35) and 41.2% (14/34) of the cases, respectively (Table 2). There was no association between CAM expression and tumor size (Table 3) or aggressive behavior (*x*^2^, *p* > 0.05). Fifteen out of 19 tumors with stage pTa were negative to p-cadherin antibody, reflecting a trend of association between stage and protein expression of this marker (*x*^2^, *p* = 0.07). A positive association was observed between e-cadherin and p-cadherin expression (*p* = 0.002) (Figure 2). The majority of the cases (25/35, 71.4%) expressed b-catenin. E-cadherin and p-cadherin positive tumors were mainly of high grade (*x*^2^, *p* = 0.037 and *p* = 0.002, resp.). P-cadherin expression was mostly found in tumors with high mitotic indices (MIB1 > 20%) (*x*^2^, *p* = 0.007). There was no association between CAM expression and muscle-invasive tumors (*x*^2^, *p* > 0.05), pointing out the small sample of the tumors examined for these adhesion molecules. Of note, a case of sarcomatoid carcinoma included in the study exhibited no immunoreactivity to antibodies for CAMs (Figure 2).

## 4. Discussion

Over the last decade, two of the HER family members, HER1/EGFR and HER2, have been researched extensively in the context of various types of cancer. Apart from their role in tumor proliferation, infiltration, and metastatic potential [22], the increasing interest in them derives from being targets of newly developed and FDA approved therapies. HER2 expression in urothelial carcinomas has been reported in several percentages ranging from 9% to 74.8% [23–25]. This discrepancy is mainly attributed to the differences in the used cutoffs and the constitution of cohorts, that is, the aggressiveness of the cases included in a study.

Notably, many studies defined HER2 overexpression as both HER2 2+ and 3+ immunoscores in urothelial carcinomas [25], as opposed to breast carcinomas. Scoring of the HercepTest corresponds to the number of extracellular domains located in the membrane [26]. In several types of cancer, such as breast carcinomas or gastric/gastroesophageal carcinomas, there are guidelines for protein expression evaluation and criteria for determining overexpression [17, 27, 28]. In urothelial carcinomas there are varying methods and cutoffs used by several studies. However, in a large cohort, Laé et al. [25] found that a true HER2 overexpression in bladder carcinomas corresponded to HER2 gene amplification, being defined in the same way as in breast cancer.

In the present study, HER2 3+ was observed in 19.6% of the cases, while HER2 2+ and 3+ account for 46.4%. The observed association between tumor grade and HER2 expression has been previously well documented [29, 30]. In addition, the aforementioned associations between HER2 expression and tumor size, pAKT, and pmTOR expression indicate that the PI3K/AKT/mTOR pathway could be activated by HER dimerization. Indeed, cases with HER2 overexpression (HER2 3+) were of high grade and they were usually characterized by pAKT and pmTOR protein overexpression and PTEN nuclear deletion. This was the immunophenotypic profile of the tumors of the two patients that presented the worst prognosis in this study. In one of these tumors, EGFR coexpression was also observed, which suggests that both AKT/PTEN/TOR and RAS/MEK/ERK pathways were activated. The increased interest to personalize therapy leads to a thorough investigation of patients who will benefit the most from a particular treatment. Hence, these patients could be the most appropriate candidates for targeted therapy, when conventional therapy fails, taking into account the side effects of these therapeutic options [31].

PTEN deletion affected more often the nucleus than the cytoplasm, a finding in line with those of previous reported studies [32, 33], and it could be found in noninfiltrating tumors but it occurs more often in muscle-invasive ones [34–36]. PTEN deletion is also observed in tumors without HER/AKT/mTOR pathway activation [35, 37], as found in our study, suggesting that PTEN loss is not always responsible for AKT activation, adding that it may be involved in other pathways [37], as it is known that different intracellular pathways are linked through cross-talking [38] or that synergistic action of different pathways is essential for carcinogenesis or aggressive biological behavior [39].

It has been reported that inactivation of p53 and inactivation of PTEN are the principal adverse prognostic markers [40]. In addition, PTEN deletion in combination with altered p53 leads to deregulation of the mTOR pathway and, consequently, reinforces the use of newly therapeutic agents, such as rapamycin [41], everolimus (RAD001) [42], or a combination of mTOR and PI3K inhibitors [43–45].

The cadherins are a group of membrane glycoprotein and the mediators of cell to cell adhesion. E-cadherin, which is an epithelial-specific cadherin, plays a major role in the selective adhesion of cells in epithelial tissue and is necessary for the maintenance of normal epithelial cells integrity. Abnormal expression of p-cadherin has been associated with an invasive and aggressive phenotype of UCs and it has been hypothesized that it may act as a key effector of muscle invasion [46]. CAMs in this study was generally coexpressed, especially in high grade tumors, but they were not associated with infiltration status, a finding that may be biased due to small sample of infiltrative tumors. As previously mentioned [46], and confirmed by this study, p-cadherin seems to be commonly expressed in high grade tumors exhibiting high mitotic index. HER pathway does not appear to affect CAM expression.

In conclusion, HER2 overexpression is found in a respective proportion of urothelial carcinomas and it seems to characterize an aggressive tumor behavior. The combination of pAKT and pmTOR expression along with a loss of PTEN expression is associated with adverse clinicopathological characteristics. P-cadherin is associated with high grade UCs but its expression is not affected by HER2 overexpression or by activation of HER/AKT/mTOR pathway.

## Figures and Tables

**Table 1:** Clinicopathologic characteristics of the tumors.

	*N*	%
pTa	22	53.6
pT1	6	14.7
pT2	9	21.9
pT3	2	4.9
pT4	2	4.9
Low grade	19	46.3
High grade	22	53.7
<3 cm	24	58.5
≥3 cm	17	41.5
One lesion	20	48.8
Multifocal	17	41.4
Missing data	4	9.8

**Table 2:** Immunohistochemical distribution of the examined markers.

	*N*	%
HER2		
0	8	19.5
1+	14	34.1
2+	11	26.8
3+	8	19.6

EGFR		
Negative	27	65.9
Positive	14	34.1

pAKT nuclear		
Negative	17	41.5
Positive	24	58.5

pAKT cytoplasmic		
Negative	29	70.7
Positive	12	29.3

PTEN cytoplasmic		
Negative	19	47.5
Positive	21	52.5
Missing data	1	—

PTEN nuclear		
Negative	31	77.5
Positive	9	22.5
Missing data	1	—

PTEN nuclear/cytoplasmic		
Negative	10	25
Positive	30	75
Missing data	1	—

pmTOR		
Negative	19	46.3
Positive	22	53.7

MIB1		
≤20	23	56.1
>20	18	43.9

e-cadherin		
Negative	16	45.7
Positive	19	54.3
Missing data	6	—

p-cadherin		
Negative	20	58.8
Positive	14	41.2
Missing data	7	—

b-catenin		
Negative	10	28.6
Positive	25	71.4
Missing data	6	—

**Table 3:** Immunoexpression of the markers according to tumors invasiveness.

	Muscle-invasive UC (pT2–pT4)	Non-muscle-invasive UC (pTa-pT1)	*p* value
HER2			
0	4	4	0.252
1	3	11	
2	2	9	
3	4	4	

EGFR			
Negative	10	17	0.308
Positive	3	11	

pAKT			
Nuclear positive	16	8	0.790
Nuclear negative	5	12	

pAKT			
Cytopl positive	2	9	0.315
Cytopl negative	11	19	

pmTOR			
Negative	9	10	**0.045**
Positive	4	18	

PTEN			
Nuclear positive	1	8	0.120
Nuclear negative	12	19	
Missing data	0	1	

PTEN			
Cytopl positive	3	18	**0.01**
Cytopl negative	10	9	
Missing data	0	1	

e-cadherin			
Negative	4	12	0.668
Positive	6	13	
Missing data	3	3	

p-cadherin			
Negative	4	16	0.307
Positive	5	9	
Missing data	4	3	

b-catenin			
Negative	2	8	0.518
Positive	8	17	
Missing data	3	3	

Cytopl: cytoplasmic; UC: urothelial carcinomas.
